# Alterations in Region‐Specific Gray Matter Volume Underlying Callous Unemotional Traits in Adolescents

**DOI:** 10.1002/brb3.70941

**Published:** 2025-09-30

**Authors:** Johannah Bashford‐Largo, Ru Zhang, R. James R. Blair, Karina S. Blair, Jaimie Elowsky, Matthew Dobbertin, Ahria J. Dominguez, Melissa Hatch, Tyler Patrick, Sahil Bajaj

**Affiliations:** ^1^ Child and Family Translational Research Center Boys Town National Research Hospital Boys Town Nebraska USA; ^2^ Center For Brain, Biology, and Behavior University of Nebraska‐Lincoln Lincoln Nebraska USA; ^3^ Weill Institute for Neurosciences University of California San Francisco California USA; ^4^ Child and Adolescent Mental Health Center Copenhagen University Hospital – Mental Health Services CPH Copenhagen Denmark; ^5^ Department of Clinical Medicine, Faculty of Health and Medical Sciences University of Copenhagen Denmark; ^6^ Center For Neurobehavioral Research Boys Town National Research Hospital Boys Town Nebraska USA; ^7^ Brain Development Laboratory Vanderbilt University Nashville Tennessee USA; ^8^ Inpatient Psychiatric Care Unit Boys Town National Research Hospital Boys Town Nebraska USA; ^9^ Clinical Health, Emotion, and Neuroscience (CHEN) Laboratory, Department of Neurological Sciences, College of Medicine University of Nebraska Medical Center (UNMC) Omaha Nebraska USA; ^10^ Cognitive and Affective Psychophysiology (CAP) Lab, Department of Psychology Saint Louis University St. Louis Missouri USA; ^11^ Department of Cancer Systems Imaging, Division of Diagnostic Imaging The University of Texas MD Anderson Cancer Center Houston Texas USA

**Keywords:** adolescents, antisocial behavior, callous‐unemotional traits, gray matter volume, magnetic resonance imaging, multiple linear regression, neurodevelopment

## Abstract

**Background:**

Callous‐unemotional (CU) traits during adolescence, for example, shallow affect or lack of remorse, have been shown to be a risk marker for antisocial behavior. Only a few studies have investigated structural brain alterations underlying CU traits, and findings are inconclusive. The study examines CU symptomatology and gray matter volume (GMV) associations.

**Methods:**

Structural brain MRI data were collected from a sample of 578 adolescents (60% male) with a mean age of 14.85 years (SD = 2.30; range = 10–19 years). CU traits were indexed via the Inventory for Callous Unemotional Traits (ICU). Region‐wise volumetric parameters were obtained following parcellation of the brain into 68 cortical and 14 subcortical regions per participant. A multiple linear regression was conducted using age, sex, IQ, intracranial volume, and handedness as covariates to assess the relationship between ICU scores and GMV.

**Results:**

Our regression analysis showed significance (*R* ^2^ = 0.244, *F*[87,490] = 1.821, *p* < 0.001). Specifically, GMV of the left parahippocampal gyrus, left pars orbitalis, right medial orbitofrontal cortex, right superior temporal, and right putamen had significant negative regression loadings, indicating those with lower GMV in these regions had higher ICU scores. The right postcentral gyrus and right hippocampus had significant positive regression loadings, indicating those with higher GMV in these regions had higher ICU scores.

**Conclusions:**

Utilizing a transdiagnostic sample of adolescents, our study found significant associations between CU traits and GMV. Understanding the neurobiological associations of CU traits could be crucial for early intervention and targeted treatments, particularly for those at risk of antisocial behavior.

## Graphical abstract

Callous‐unemotional (CU) traits in adolescence indicate risk for antisocial behavior. Few studies explore structural brain alterations related to CU traits, leading to inconclusive findings. We analyzed MRI data from 578 adolescents (mean age 14.85) to assess the relationship between CU traits and GMV. Significant regression (*R* ^2^ = 0.494, *p* < 0.001) indicated that lower GMV in specific brain regions correlated with higher CU scores. Our findings highlight the importance of understanding the neurobiological links of CU traits for early intervention in antisocial behavior.

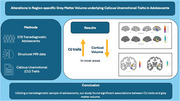

## 1 Introduction

Callous‐unemotional (CU) traits reflect a lack of empathy, guilt, and an indifference to the individual's actions and a lack of concern for one's own or others' feelings (Frick et al. 2014). The DSM‐5 includes a specifier for conduct disorder (CD), called limited prosocial emotions, that captures these traits (APA 2022). Adolescents with CU traits are highly susceptible to more severe, chronic, and treatment‐resistant forms of CD (Frick et al. 2014) and later psychopathic traits in adulthood (Burke et al. 2007; Salekin and Frick 2005; Vasey et al. 2005). Understanding the behavioral and neural components of CU traits in childhood is key for early intervention.

Neuroimaging studies implicate dysfunction across cortical and subcortical regions, including the amygdala, insula, ventromedial prefrontal cortex (vmPFC), and frontal and temporal cortices, in youth with CU traits (Blair 2019; Hwang et al. 2016; Viding et al. 2012; Zhang, Aloi, et al. 2021). Structural brain abnormalities, specifically alterations in gray matter volume (GMV), have been identified in individuals with CU traits. GMV alterations in frontal regions may contribute to atypical emotional processing and impulse control (De Brito et al. 2021; Sakki et al. 2023; Sebastian et al. 2014). Findings, however, remain inconsistent. Some studies report smaller orbitofrontal cortex (OFC), anterior cingulate cortex (ACC), and middle frontal gyrus volumes (Sebastian et al. 2016; Tkalcec et al. 2023), while others observe larger or asymmetric volumes (De Brito et al. 2009; Fairchild et al. 2013; Lam et al. 2021). Many of these studies used small samples (*N* = 29–130) and varied CU trait measures (Antisocial Process Screening Device vs. Inventory of CU Traits, using self‐ vs. parent/teacher‐report), which may contribute to inconsistent frontal findings.

Temporal and parietal findings also vary, with both positive and negative associations reported for the superior temporal gyrus, anterior temporal lobe, and parietal lobules (Caldwell et al. 2019; De Brito et al. 2009). Studies examining the insula report both positive (Cohn et al. 2016; Raschle et al. 2015) and negative (Fairchild et al. 2013) associations, with the latter specifically in females. Subcortical findings also show similarly mixed results. Higher CU traits have been linked to smaller amygdala and putamen volumes in some studies (Cardinale et al. 2019; Fairchild et al. 2013; Gao et al. 2020; Rogers and De Brito 2016), but other work has found larger hippocampal or striatal volumes (De Brito et al. 2009; Jiang et al. 2023).

To address these limitations, the present study examines cortical and subcortical GMV in a large, transdiagnostic adolescent sample that includes youth with and without psychiatric diagnoses. This approach improves statistical power, accounts for comorbidities, and allows for dimensional analyses of CU traits. Analyses controlled for demographic and cognitive factors known to influence morphometry, including age, sex, IQ, intracranial volume (ICV), and handedness (Jang et al. 2017; Sakki et al. 2023; Shiner and Caspi 2003). We hypothesized that higher CU traits would be associated with reduced GMV in frontal regions (OFC, ACC, frontal gyri) and temporal regions, given their roles in emotion regulation and executive functioning, and with smaller amygdala volumes based on prior evidence. Associations with the hippocampus and striatum were explored because prior studies have reported inconsistent findings. Notably, this study complements recent large‐scale efforts such as the ENIGMA‐Antisocial Behavior Working Group, which identified overlapping regions involved in externalizing traits across development (Gao et al. 2024). By integrating dimensional CU measures in a transdiagnostic youth sample, our findings aim to clarify neuroanatomical correlates of CU traits and guide future biomarker‐informed research.

## 2 Methods

### 2.1 Participants

For this study 578 adolescents aged between 10 and 19 years from a residential program and the surrounding town were recruited. Reasons for recruitment to the program included referral for mental health or behavioral issues. Recruitment was done through flyers and social media for participants outside of the program.

A licensed, board‐certified psychiatrist completed clinical characterization with the youth and their guardians. The Boys Town National Research Hospital (BTNRH) institutional review board approved this study. Written consent and assent were obtained by a doctoral‐level researcher. Participants were reminded that they had the right to decline participation in the study.

Some exclusion criteria included having an IQ of < 75 (via Wechsler Abbreviated Scale of Intelligence [Wechsler 2011]), psychosis, neurological disorders, presence of metal in the body, and claustrophobia. We selected an IQ cutoff of 75 to exclude participants with intellectual disability while retaining those with borderline or subclinical cognitive functioning, preserving ecological validity and ensuring sufficient cognitive ability for valid task performance. See previous papers for full methods (Bashford‐Largo, Blair, et al. 2023; Zhang et al. 2023).

Some behavioral data from this sample (e.g., including measures of suicide risk, irritability, aggression, CD, depression, and anxiety) and related morphometric characteristics have been previously reported (Bajaj et al. 2021, 2023; Bashford‐Largo et al. 2022, 2023, 2024); however, the associations between ICU and brain morphometry are novel and have not been published before.

### 2.2 Data Collection

#### 2.2.1 Neuroanatomical Data

A 3‐T MRI scanner (Siemens Prisma) was used to obtain T1‐weighted data. Participants were instructed to rest, relax, and minimize head movement throughout the scan. A 3D MPRAGE sequence was used to capture high‐resolution anatomical images of the whole brain, which consisted of 176 axial slices (thickness of 1 mm) and voxel resolution of 0.9 × 0.9 × 1 mm^3^. The sequence parameters included a repetition time of 2200 ms, an echo time of 2.48 ms, a matrix size of 256 × 208, a FOV of 230 mm, and a flip angle of 8° (same as previous papers [Bashford‐Largo, Nakua, et al. 2023; Zhang, Aloi, et al. 2021]).

#### 2.2.2 Measures

*Inventory of Callous‐Unemotional Traits (ICU)*: The ICU (Frick 2004) is a 24‐item self‐report questionnaire designed to assess CU traits in youth. Each item is rated on a 4‐point Likert scale (0 = *not at all true* to 3 = *definitely true*), and the measure yields a total score as well as three subscales: Callousness (CA), Uncaring (UC), and Unemotional (UE). The ICU has demonstrated good construct validity and internal consistency (coefficient alpha ≈ 0.77) in both community and juvenile justice populations (Essau et al. 2006; Kimonis et al. 2008).

*WASI‐II*: IQ was estimated using the Wechsler Abbreviated Scale of Intelligence, Second Edition (WASI‐II; Wechsler 2011), which assesses perceptual reasoning, verbal comprehension, and Full‐Scale IQ (FSIQ). FSIQ scores demonstrate excellent reliability (*α* = 0.98) and correlate strongly with scores on the full Wechsler Adult Intelligence Scale (WAIS‐III; *r* = 0.92; Wechsler 1997, 1999), supporting their use in this study.

### 2.3 Image Preprocessing

The processing of anatomical brain images (Dale et al. 1999) and estimating of GMV were done by *recon‐all* from FreeSurfer (Version 6.0). Structural image preprocessing involved multiple stages, including motion correction, skull stripping, spatial normalization to the MNI template, segmentation of cortical and subcortical structures, intensity calibration, and cortical parcellation into gyral and sulcal regions (Desikan et al. 2006). See previous publications for further details (Dale et al. 1999; Fischl 2004; Fischl et al. 1999). Quality assurance was ensured through a careful visual inspection (see previous papers [Bashford‐Largo et al. 2022]).

### 2.4 Data Analysis

The brain was segmented into distinct regions using the Desikan atlas (Desikan et al. 2006), partitioning the cortex into 68 areas (34 per hemisphere) along with 14 subcortical structures (i.e., seven regions for each hemisphere) using the default parcellation scheme implemented within FreeSurfer. GMV data from these 68 cortical as well as 14 subcortical regions (i.e., Putamen, Pallidum, Hippocampus, Thalamus, Amygdala, Caudate, and Accumbens area) were evaluated and exported for the left and the right hemispheres separately. This process utilized FreeSurfer's *recon‐all, mri_surf2surf, mris_anatomical_stats*, and *aparcstats2table* for surface‐based analysis and anatomical measurements (Fischl et al. 1999).

A multiple linear regression analysis (*α* = 0.05, two‐tailed) was conducted to predict CU scores, using volumes from 68 cortical and 14 subcortical regions as independent variables. Age, IQ, sex, ICV, and handedness (assessed via self‐report and coded as a binary variable) were used as covariates. Our decision to model age linearly is consistent with prior evidence that cortical GMV largely declines in a linear fashion during adolescence (Tamnes et al. 2017), allowing us to account for developmental effects without overfitting. Data were analyzed within IBM SPSS (version 25) software (IBM Corp. 2019) using only participants with complete data.

### 2.5 Follow‐Up Analyses

Given that many of the adolescents had psychiatric diagnoses and were on psychotropic medication (see Table 1), a follow‐up regression including the seven diagnoses (MDD, GAD, SAD, ADHD, CD, and ODD) and three types of medication (antipsychotics, stimulants, and SSRIs) was run to explore the possible effects of these confounding variables (*N* = 578).

**TABLE 1:** Demographics of participants (*N* = 578).

Mean age	14.85 (SD = 2.30)
Sex	230 F/348 M
Mean IQ	103.45 (SD = 3.08)
Handedness	511 (88.4%) right, 67 (11.6%) left
Mean ICU total score	21.35 (SD = 8.73) (range 3–51; possible 0–72)
Race	
Native American	12 (2.1%)
Native Hawaiian or other Pacific Islander	1 (0.2%)
Asian	6 (1.0%)
Black or African American	59 (10.2%)
White	447 (77.3%)
Multiracial	60 (10.4%)
Not reported	21 (3.6%)
Ethnicity	
Hispanic	41 (7.1%)
Non‐Hispanic	497 (86.0%)
Not reported	40 (6.9%)
Psychiatric Dxs	
MDD	52 (9.0%)
SAD	110 (19.0%)
GAD	104 (18.0%)
PTSD	51 (8.8%)
CD	200 (34.6%)
ADHD	269 (46.5%)
ODD	252 (43.6%)
No diagnosis	216 (37.4%)
Medication	
Antipsychotics	32 (5.5%)
Stimulants	88 (15.2%)
SSRIs	62 (10.7%)

Abbreviations: ADHD, attention‐deficit/hyperactivity disorder; CD, conduct disorder; GAD, generalized anxiety disorder; ICU, inventory of callous–unemotional traits; MDD, major depressive disorder; ODD, oppositional defiant disorder; PTSD, post‐traumatic stress disorder; SAD, social anxiety disorder; SSRIs, selective serotonin reuptake inhibitors.

## 3 Results

### 3.1 Participant Sample

A total of 578 participants were included in this study (mean age = 14.85 ± 2.30 years; 60.2% male). The average IQ was 103.45 ± 3.08, and most were right‐handed (511; 88.4%). The mean ICU total score was 21.35 ± 8.73 (range = 3–51; possible = 0–72) (see Table 2 for full demographics). Only participants with full data were included in the study.

**TABLE 2:** Significant brain regions predicting ICU total scores in the full regression model.

Region	Unstandardized beta	Std. error	Standardized beta	*t*	*p*‐value
LH Parahippocampal Gyrus	−0.003	0.001	−0.133	−2.218	0.027
LH Pars Orbitalis	−0.003	0.001	−0.153	−2.292	0.022
RH Medial Orbitofrontal	−0.002	0.001	−0.226	−2.868	0.004
RH Superior Temporal	−0.001	0.000	−0.279	−2.992	0.003
RH Postcentral Gyrus	0.001	0.000	0.166	2.232	0.026
RH Hippocampus	0.005	0.002	0.233	2.645	0.008
RH Putamen	−0.004	0.002	−0.277	−2.075	0.038

*Note*: Negative beta values indicate that lower gray matter volume in a region was associated with higher ICU scores, while positive beta values indicate the opposite.

Abbreviations: LH, left hemisphere; RH, right hemisphere.

**FIGURE 1:**
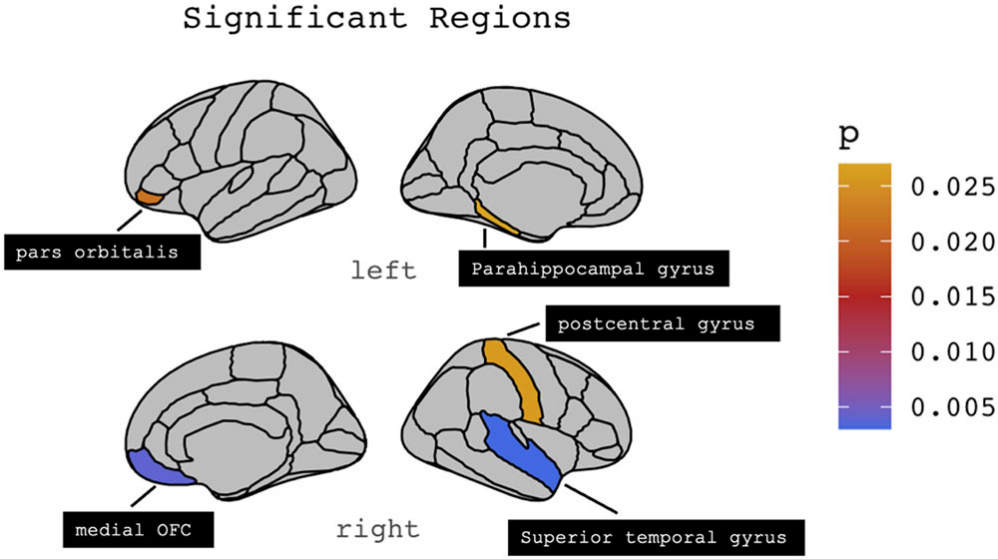
Cortical findings: For cortical regions, we had significant negative loadings within the left pars orbitalis (*B* = −0.153, *p* = 0.022), left parahippocampal gyrus (*B* = −0.133, *p* = 0.027), right medial orbitofrontal cortex (*B* = −0.226, *p* = 0.004), and right superior temporal cortex (*B* = −0.279, *p* = 0.003). The right postcentral gyrus (*B* = 0.166, *p* = 0.026) had positive loadings.

**FIGURE 2:**
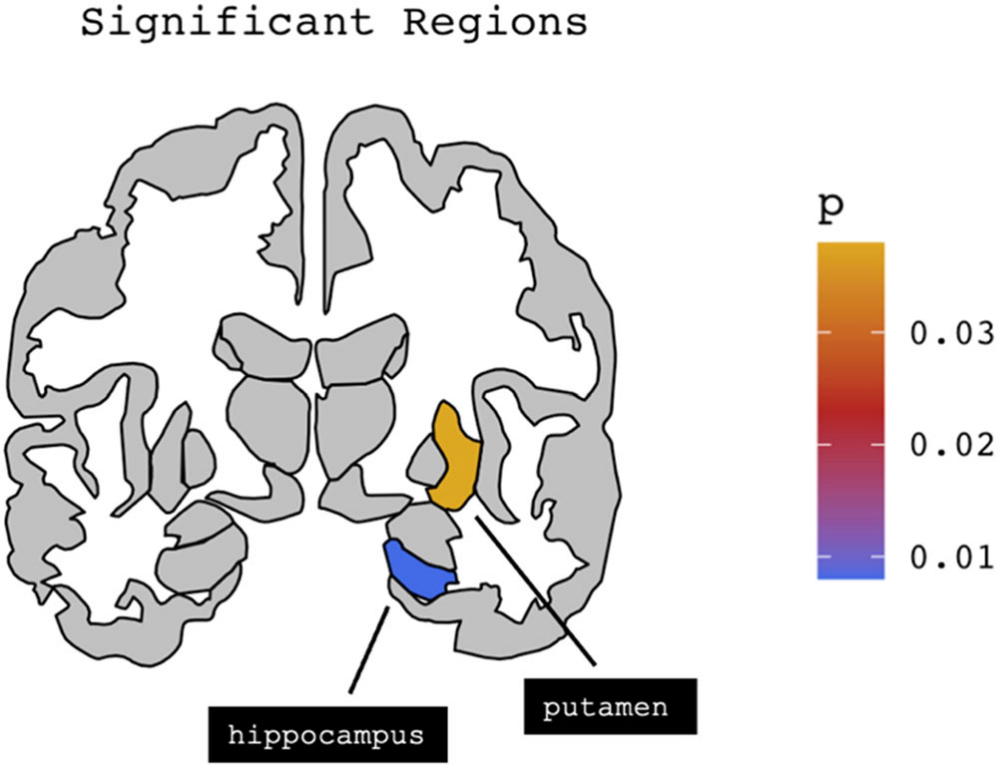
Subcortical findings: For subcortical regions, we had significant negative loadings within right putamen (*B* = −0.277, *p* = 0.038) and positive loadings within right hippocampus (*B* = 0.233, *p* = 0.008).

Regression assumptions were evaluated and met. Residual plots supported linearity and homoscedasticity, and residuals were approximately normally distributed. The Durbin‐Watson statistic (1.70) indicated independence of errors. VIF values were generally acceptable, though a few exceeded conservative thresholds, likely due to correlations among brain regions. Our regression analysis yielded a significant model (*R* ^2^ = 0.244, *F*(87,490) = 1.821, *p* < 0.001, *f^2^* = 0.323) with significant negative loadings within the left pars orbitalis (*B* = −0.153, *p* = 0.022), left parahippocampal (*B* = −0.133, *p* = 0.027), right medial orbitofrontal cortex (*B* = −0.226, *p* = 0.004), right superior temporal (*B* = −0.279, *p* = 0.003) and right putamen (*B* = −0.277, *p* = 0.038). The right postcentral gyrus (*B* = 0.166, *p* = 0.026) and right hippocampus (*B* = 0.233, *p* = 0.008) had positive loadings (Figures 1 and 2).

### 3.2 Follow‐Up Analyses

Follow‐up regression analysis with psychiatric diagnoses and medications mirrored results of the main analysis (*R* ^2^ = 0.343, *F*(97,480) = 2.583, *p* < 0.001, *f* ^2^ = 0.522) except for the significance of the right postcentral gyrus (*p* = 0.057), indicating that including psychiatric diagnoses and medications did not significantly change the majority of the main results.Full results are provided in the Supplementary Information.

## 4 Discussion

This study aimed to assess the relationship between GMV and CU traits. The main results of this study showed GMV was *negatively* associated with CU symptomatology within multiple frontal, temporal, and subcortical regions.

The frontal lobe is critical for higher‐order cognitive functions, such as decision‐making and emotional regulation. The abnormalities within this region may limit the capacity of modulating emotion due to deficient limbic‐frontal communications (Banks et al. 2007). Dysfunctional brain activities have been previously identified in CU adolescents in middle frontal gyrus (Cardinale et al. 2018; Marsh et al. 2008; Murray et al. 2023; Rhoads et al. 2020), ACC (Hawes et al. 2021; Michalska et al. 2016; Rhoads et al. 2020; Sebastian et al. 2014; Winters et al. 2021), medial prefrontal cortex (Rhoads et al. 2020; Veroude et al. 2016; Zhang, Aloi, et al. 2021), frontal pole (Rhoads et al. 2020), pars triangularis (Rhoads et al. 2020), OFC (Hawes et al. 2021), and superior frontal gyrus (Hawes et al. 2021; Rhoads et al. 2020). The components within the frontal lobe associated with reduced GMV in the current study included right medial OFC. The structural alteration in OFC aligned with Sebastian et al.'s (2016) findings of smaller OFC in participants with conduct problems and higher CU traits versus participants with conduct problems and lower CU traits, and the controls. However, this has been contradicted in a study done by Fairchild et al. (2013) in which positive correlations between CU traits and GMV in this region were observed. Methodological differences likely explain inconsistent OFC findings across studies. Variability in age ranges (child vs. adolescent samples), sex distribution, CU trait measures (parent vs. self‐report), comorbid diagnoses, MRI processing methods (voxel‐based vs. surface‐based morphometry), and small sample sizes (< 100) can all influence GMV estimates. These factors may account for reports of both increased and decreased OFC volume in relation to CU traits. Structural alterations within these areas are particularly important for understanding CU traits due to the emotional and executive functions of the frontal lobe, which can significantly impact behavior. For instance, structural differences within the OFC may contribute to difficulties in responding appropriately to emotional and social cues, which are core characteristics of CU traits.

Previous studies show associations with CU traits and abnormal brain activity in temporal lobe regions (Cardinale et al. 2018; Fairchild et al. 2015; Murray et al. 2023; Rhoads et al. 2020; Werhahn et al. 2023). In the current study, the volume of left pars orbitalis, left parahippocampal gyrus, and right superior temporal gyrus had negative loadings. With respect to superior temporal gyrus, De Brito et al. (2009) found the size of this region greater for the boys with conduct problems and CU traits relative to the controls.

We found two subcortical regions significantly contributing to the model: the right putamen and right hippocampus. This finding was consistent with a previous study showing reductions in GMV in the right putamen within individuals with CD (Fairchild et al. 2013). As the putamen is part of ventral striatum, it is implicated in integration of stimulus, response, and outcome (Yamada et al. 2004). Specifically, putamen activation has been shown to be correlated with reward magnitude (Cromwell and Schultz 2003) and errors in predicting the timing of reward (McClure et al. 2003). Altered structure of putamen observed in the current study mirrored finding of the functional MRI study in which CU traits were associated with dysfunctional reinforcement learning processing in striatum (Schwenck et al. 2017). Structural changes within the putamen may underlie the atypical reward sensitivity often associated with CU traits (Zhang, Aloi, et al. 2021). Results within the hippocampus have been mixed. For instance, one study found larger hippocampal volumes associated with CU traits in boys (De Brito et al. 2009). However, high CU traits have also been linked with smaller hippocampal volumes (Jiang et al. 2023; Waller et al. 2020). In the current study, larger hippocampal subcortical volume was associated with higher CU traits. The increased GMV in the right hippocampus and postcentral gyrus may reflect atypical neurodevelopment. This could involve delayed synaptic pruning, altered maturation rates, or compensatory plasticity, which are mechanisms that may lead to transient increases in volume during adolescence. For example, increased hippocampal volume could reflect a compensatory response to reduced regulation from frontal regions (Selemon 2013; Sheth et al. 2017). These mixed results highlight the complexity of CU traits in relation to structural differences within the hippocampus and could indicate that there is influence by additional factors such as an individual's developmental stage or sex (Gennatas et al. 2017; Raznahan et al. 2010). These regions may also serve as potential biomarkers to guide targeted interventions. For example, hippocampal involvement suggests that memory‐ and context‐based processes are relevant intervention targets, while frontal regions implicated in CU traits highlight the importance of improving top‐down control of affective responses.

An important caveat regarding the current results is the cross‐sectional design, which prevents conclusions about causality, as well as the potential influence of medications and psychiatric diagnoses on participants. Some participants took medications (i.e., SSRIs, antipsychotic medications, or stimulants) and/or were diagnosed with psychiatric conditions. Although these factors could have affected the results, our follow‐up analyses indicated results were proximal to the main analysis.

In conclusion, this study leveraged a large, transdiagnostic sample of adolescents and a dimensional assessment of CU traits to examine cortical and subcortical gray matter associations. We found structural alterations in frontal, temporal, and subcortical regions linked to CU traits. These findings highlight potential neural correlates of CU traits in youth and underscore the value of large‐scale, dimensional approaches for identifying brain‐behavior relationships. To build on these findings, future longitudinal studies are needed to examine how structural brain differences develop over time in relation to CU traits. Such studies could clarify whether these neuroanatomical differences emerge early and persist, reflect delayed maturation, or evolve alongside behavioral symptoms. Repeated neuroimaging across adolescence would also help determine whether these structural features are stable markers of CU traits or responsive to environmental or therapeutic interventions).

## Author Contributions

R. James R. Blair contributed to conceptualization, methodology, supervision, funding acquisition, visualization, and project administration. Karina S. Blair contributed to conceptualization, methodology, and supervision. Sahil Bajaj contributed to methodology, data curation, formal analysis, supervision, and project administration. Johannah Bashford‐Largo contributed to formal analysis and writing, original draft, as well as writing, review and editing. Ru Zhang contributed to formal analysis and writing, original draft, as well as writing, review, and editing. Jaimie Elowsky contributed to investigation and writing, review and editing. Matthew Dobbertin contributed to investigation, validation, supervision, and writing, review and editing. Ahria J. Dominguez contributed to data curation and writing, review and editing. Melissa Hatch contributed to data curation and writing, review and editing. Tyler Patrick contributed to supervision and writing, review and editing.

## Ethics Statement

The authors confirm that all procedures involved in this work adhere to the ethical guidelines set forth by the appropriate national and institutional committees for human experimentation, in accordance with the Helsinki Declaration of 1975.

## Conflicts of Interest

The authors declare no conflicts of interest.

## Peer Review

The peer review history for this article is available at https://publons.com/publon/10.1002/brb3.70941

## Supporting information




**Supplementary Material**: brb370941‐sup‐0001‐SuppMatt.docx

## Data Availability

The data that support the findings of this study are available on request from the corresponding author. The data are not publicly available due to privacy or ethical restrictions.
