# Supplementary material for: Alterations in Region‐Specific Gray Matter Volume Underlying Callous Unemotional Traits in Adolescents
Source: Brain Behav. 2025 Sep 30;15(10):e70941. doi: 10.1002/brb3.70941 (PMC12480921; doi:10.1002/brb3.70941)
Supplement: Supplementary file 1 — Supplementary Material: brb370941‐sup‐0001‐SuppMatt.docx [file BRB3-15-e70941-s001.docx]

**SUPPLEMENTAL MATERIAL**

**IQ Analysis**

To assess the impact of IQ thresholding, we re-ran the analysis using a more conservative cutoff of IQ ≥ 80. The multiple linear regression model significantly predicted ICU total scores, R² = .235, F(87, 474) = 1.673, p < .001. Several brain regions emerged as significant: lower volumes in the left parahippocampal gyrus, left pars orbitalis, right medial orbitofrontal cortex, right superior temporal gyrus, left hippocampus, and right putamen were associated with higher ICU scores. In contrast, greater volumes in the right postcentral gyrus and right hippocampus were associated with higher ICU scores. No regions lost significance from the main model.



| **Region** | **Hemisphere** | **Standardized Beta (β)** | **p-value** |
| --- | --- | --- | --- |
| Parahippocampal Gyrus | Left | –.124 | .044 |
| Pars Orbitalis | Left | –.152 | .028 |
| Medial Orbitofrontal Cortex | Right | –.193 | .017 |
| Superior Temporal Gyrus | Right | –.281 | .003 |
| Hippocampus | Left | –.187 | .040 |
| Putamen | Right | –.284 | .036 |
| Postcentral Gyrus | Right | .165 | .029 |
| Hippocampus | Right | .226 | .012 |

**Medication Analysis**

When youth taking psychotropic medications (antipsychotics, stimulants, SSRIs) were excluded, the regression model predicting ICU total scores remained significant, *R²* = .28, *F*(87, 352) = 1.57, *p* = .002. Several regions that also emerged in the full sample were significant predictors. Specifically, higher ICU scores were associated with reduced volume in the left parahippocampal gyrus (β = -.16, *p* = .023), left pars orbitalis (β = -.16, *p* = .045), right superior temporal gyrus (β = -.37, *p* < .001), and right medial orbitofrontal cortex (β = -.20, *p* = .034). In contrast, greater ICU scores were associated with increased volume in the right postcentral gyrus (β = .30, *p* < .001).

Supplementary Table 1 presents the full multiple linear regression output predicting ICU total scores. The table includes unstandardized (B) and standardized (β) coefficients, standard errors, t-values, and p-values for all predictors included in the model.

| Variable | B | Std. Error | Beta | t | Sig. |
| --- | --- | --- | --- | --- | --- |
| (Constant) | 35.774 | 7.298 |  | 4.902 | <.001 |
| sex_v2___1 | 2.677 | 1.005 | 0.15 | 2.664 | 0.008 |
| sum_of_t_scores | -0.124 | 0.031 | -0.186 | -4.043 | <.001 |
| handedness_v2 | -1.541 | 1.195 | -0.057 | -1.289 | 0.198 |
| Age | 0.332 | 0.228 | 0.088 | 1.458 | 0.145 |
| lh_bankssts_volume | 0.001 | 0.001 | 0.058 | 0.919 | 0.359 |
| lh_caudalanteriorcingulate_volume | 0.001 | 0.001 | 0.029 | 0.507 | 0.612 |
| lh_caudalmiddlefrontal_volume | 0.0 | 0.0 | -0.074 | -1.106 | 0.269 |
| lh_cuneus_volume | 0.0 | 0.001 | 0.01 | 0.125 | 0.9 |
| lh_entorhinal_volume | 0.001 | 0.001 | 0.049 | 0.873 | 0.383 |
| lh_fusiform_volume | -5.93e-05 | 0.0 | -0.01 | -0.137 | 0.891 |
| lh_inferiorparietal_volume | 0.0 | 0.0 | -0.119 | -1.491 | 0.137 |
| lh_inferiortemporal_volume | 0.0 | 0.0 | 0.119 | 1.454 | 0.147 |
| lh_isthmuscingulate_volume | -0.001 | 0.001 | -0.064 | -0.988 | 0.323 |
| lh_lateraloccipital_volume | 0.0 | 0.0 | 0.049 | 0.644 | 0.52 |
| lh_lateralorbitofrontal_volume | 0.001 | 0.001 | 0.193 | 1.824 | 0.069 |
| lh_lingual_volume | 0.0 | 0.001 | 0.051 | 0.685 | 0.494 |
| lh_medialorbitofrontal_volume | 0.001 | 0.001 | 0.057 | 0.818 | 0.414 |
| lh_middletemporal_volume | -0.001 | 0.0 | -0.126 | -1.508 | 0.132 |
| lh_parahippocampal_volume | -0.003 | 0.002 | -0.133 | -2.218 | 0.027 |
| lh_paracentral_volume | -0.001 | 0.001 | -0.058 | -0.955 | 0.34 |
| lh_parsopercularis_volume | 2.935e-05 | 0.0 | 0.004 | 0.061 | 0.951 |
| lh_parsorbitalis_volume | -0.003 | 0.001 | -0.153 | -2.292 | 0.022 |
| lh_parstriangularis_volume | 0.0 | 0.001 | -0.024 | -0.374 | 0.709 |
| lh_pericalcarine_volume | 0.0 | 0.002 | 0.017 | 0.175 | 0.861 |
| lh_postcentral_volume | 3.858e-05 | 0.0 | 0.007 | 0.094 | 0.925 |
| lh_posteriorcingulate_volume | 0.001 | 0.001 | 0.098 | 1.646 | 0.1 |
| lh_precentral_volume | 0.0 | 0.0 | -0.029 | -0.342 | 0.733 |
| lh_precuneus_volume | 0.0 | 0.0 | 0.023 | 0.262 | 0.794 |
| lh_rostralanteriorcingulate_volume | 0.001 | 0.001 | 0.074 | 1.041 | 0.298 |
| lh_rostralmiddlefrontal_volume | 0.0 | 0.0 | -0.091 | -0.993 | 0.321 |
| lh_superiorfrontal_volume | 4.79e-05 | 0.0 | 0.018 | 0.178 | 0.859 |
| lh_superiorparietal_volume | -7.69e-05 | 0.0 | -0.02 | -0.251 | 0.802 |
| lh_superiortemporal_volume | 0.001 | 0.0 | 0.144 | 1.706 | 0.089 |
| lh_supramarginal_volume | -6.223e-05 | 0.0 | -0.017 | -0.26 | 0.795 |
| lh_frontalpole_volume | 0.0 | 0.002 | 0.004 | 0.075 | 0.94 |
| lh_temporalpole_volume | 0.001 | 0.001 | 0.036 | 0.681 | 0.496 |
| lh_transversetemporal_volume | 0.002 | 0.002 | 0.05 | 0.833 | 0.405 |
| lh_insula_volume | -0.001 | 0.001 | -0.083 | -0.944 | 0.346 |
| rh_bankssts_volume | 0.001 | 0.001 | 0.075 | 1.211 | 0.227 |
| rh_caudalanteriorcingulate_volume | 4.498e-05 | 0.001 | 0.003 | 0.042 | 0.966 |
| rh_caudalmiddlefrontal_volume | -5.547e-05 | 0.0 | -0.008 | -0.127 | 0.899 |
| rh_cuneus_volume | -0.001 | 0.001 | -0.093 | -1.241 | 0.215 |
| rh_entorhinal_volume | -0.002 | 0.001 | -0.101 | -1.866 | 0.063 |
| rh_fusiform_volume | 0.0 | 0.0 | -0.051 | -0.662 | 0.508 |
| rh_inferiorparietal_volume | -4.54e-05 | 0.0 | -0.016 | -0.204 | 0.838 |
| rh_inferiortemporal_volume | -2.533e-05 | 0.0 | -0.006 | -0.073 | 0.942 |
| rh_isthmuscingulate_volume | 0.0 | 0.001 | 0.014 | 0.214 | 0.83 |
| rh_lateraloccipital_volume | 0.0 | 0.0 | -0.081 | -1.015 | 0.311 |
| rh_lateralorbitofrontal_volume | 9.859e-05 | 0.001 | 0.015 | 0.138 | 0.89 |
| rh_lingual_volume | -3.008e-05 | 0.0 | -0.005 | -0.066 | 0.948 |
| rh_medialorbitofrontal_volume | -0.002 | 0.001 | -0.226 | -2.868 | 0.004 |
| rh_middletemporal_volume | 0.0 | 0.0 | 0.065 | 0.699 | 0.485 |
| rh_parahippocampal_volume | 0.003 | 0.002 | 0.111 | 1.922 | 0.055 |
| rh_paracentral_volume | 0.002 | 0.001 | 0.121 | 1.965 | 0.05 |
| rh_parsopercularis_volume | 0.0 | 0.001 | 0.038 | 0.596 | 0.551 |
| rh_parsorbitalis_volume | 0.0 | 0.001 | 0.016 | 0.24 | 0.811 |
| rh_parstriangularis_volume | 0.001 | 0.001 | 0.076 | 1.076 | 0.282 |
| rh_pericalcarine_volume | 0.0 | 0.002 | 0.017 | 0.182 | 0.855 |
| rh_postcentral_volume | 0.001 | 0.0 | 0.166 | 2.232 | 0.026 |
| rh_posteriorcingulate_volume | 0.0 | 0.001 | -0.029 | -0.436 | 0.663 |
| rh_precentral_volume | 0.0 | 0.0 | -0.031 | -0.366 | 0.714 |
| rh_precuneus_volume | 0.0 | 0.0 | -0.077 | -0.833 | 0.405 |
| rh_rostralanteriorcingulate_volume | 0.001 | 0.001 | 0.037 | 0.572 | 0.568 |
| rh_rostralmiddlefrontal_volume | 0.0 | 0.0 | 0.152 | 1.574 | 0.116 |
| rh_superiorfrontal_volume | 0.0 | 0.0 | -0.074 | -0.738 | 0.461 |
| rh_superiorparietal_volume | 0.0 | 0.0 | 0.111 | 1.278 | 0.202 |
| rh_superiortemporal_volume | -0.001 | 0.0 | -0.279 | -2.992 | 0.003 |
| rh_supramarginal_volume | 0.0 | 0.0 | 0.085 | 1.123 | 0.262 |
| rh_frontalpole_volume | -0.003 | 0.002 | -0.08 | -1.408 | 0.16 |
| rh_temporalpole_volume | -0.001 | 0.001 | -0.044 | -0.832 | 0.406 |
| rh_transversetemporal_volume | 2.76e-05 | 0.003 | 0.001 | 0.009 | 0.993 |
| rh_insula_volume | 0.0 | 0.001 | -0.027 | -0.316 | 0.752 |
| eTIV | 8.005e-07 | 0.0 | 0.014 | 0.135 | 0.893 |
| LeftThalamus | 0.0 | 0.001 | -0.013 | -0.105 | 0.917 |
| Left-Caudate | -0.001 | 0.003 | -0.079 | -0.539 | 0.59 |
| Left-Putamen | 0.002 | 0.002 | 0.125 | 0.951 | 0.342 |
| Left-Pallidum | 0.003 | 0.003 | 0.093 | 1.07 | 0.285 |
| Left-Hippocampus | -0.004 | 0.002 | -0.17 | -1.931 | 0.054 |
| Left-Amygdala | 0.003 | 0.003 | 0.074 | 0.925 | 0.355 |
| Left-Accumbens-area | -0.003 | 0.006 | -0.04 | -0.56 | 0.576 |
| RightThalamus | 0.0 | 0.001 | -0.039 | -0.314 | 0.754 |
| Right-Caudate | 0.004 | 0.003 | 0.21 | 1.349 | 0.178 |
| Right-Putamen | -0.004 | 0.002 | -0.277 | -2.075 | 0.038 |
| Right-Pallidum | 0.0 | 0.003 | 0.012 | 0.128 | 0.899 |
| Right-Hippocampus | 0.005 | 0.002 | 0.233 | 2.645 | 0.008 |
| Right-Amygdala | -0.003 | 0.003 | -0.08 | -1.01 | 0.313 |
| Right-Accumbens-area | 0.0 | 0.006 | -0.002 | -0.034 | 0.973 |

Key: lh = left hemisphere, rh = right hemisphere
